# *N*-(3-Chloro-1*H*-indazol-5-yl)-4-meth­oxy­benzene­sulfonamide

**DOI:** 10.1107/S160053681302744X

**Published:** 2013-10-12

**Authors:** Hakima Chicha, El Mostapha Rakib, Latifa Bouissane, Mohamed Saadi, Lahcen El Ammari

**Affiliations:** a Laboratoire de Chimie Organique et Analytique, Université Sultan Moulay Slimane, Faculté des Sciences et Techniques, Béni-Mellal, BP 523, Morocco; b Laboratoire de Chimie du Solide Appliquée, Faculté des Sciences, Université Mohammed V-Agdal, Avenue Ibn Battouta, BP 1014, Rabat, Morocco

## Abstract

In the title compound, C_14_H_12_ClN_3_O_3_S, the fused five- and six-membered rings are folded slightly along the common edge, forming a dihedral angle of 3.2 (1)°. The mean plane through the indazole system makes a dihedral angle of 30.75 (7)° with the distant benzene ring. In the crystal, N—H⋯O hydrogen bonds link the mol­ecules, forming a two-dimensional network parallel to (001).

## Related literature

For the pharmacological activity of sulfonamide derivatives, see: Bouissane *et al.* (2006 ▶); El-Sayed *et al.* (2011 ▶); Mustafa *et al.* (2012 ▶). For similar compounds, see: Abbassi *et al.* (2012 ▶, 2013 ▶); Chicha *et al.* (2013 ▶).
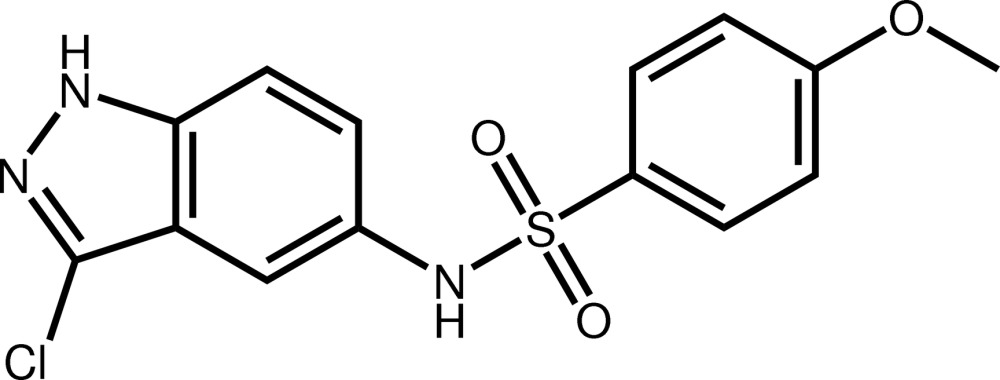


## Experimental

### 

#### Crystal data

C_14_H_12_ClN_3_O_3_S
*M* *_r_* = 337.78
Monoclinic, 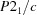

*a* = 16.1229 (5) Å
*b* = 10.0562 (3) Å
*c* = 9.7955 (2) Å
β = 105.388 (1)°
*V* = 1531.26 (7) Å^3^
*Z* = 4
Mo *K*α radiation
μ = 0.40 mm^−1^
*T* = 296 K
0.42 × 0.35 × 0.28 mm

#### Data collection

Bruker X8 APEX diffractometer
Absorption correction: multi-scan (*SADABS*; Bruker, 2009 ▶) *T*_min_ = 0.693, *T*_max_ = 0.747
19939 measured reflections
4666 independent reflections
3973 reflections with *I* > 2σ(*I*)
*R*_int_ = 0.022

#### Refinement

*R*[*F* ^2^ > 2σ(*F* ^2^)] = 0.040
*wR*(*F* ^2^) = 0.117
*S* = 1.03
4666 reflections
199 parameters
H-atom parameters constrained
Δρ_max_ = 0.63 e Å^−3^
Δρ_min_ = −0.49 e Å^−3^

### 

Data collection: *APEX2* (Bruker, 2009 ▶); cell refinement: *SAINT* (Bruker, 2009 ▶); data reduction: *SAINT*; program(s) used to solve structure: *SHELXS97* (Sheldrick, 2008 ▶); program(s) used to refine structure: *SHELXL97* (Sheldrick, 2008 ▶); molecular graphics: *ORTEP-3 for Windows* (Farrugia, 2012 ▶); software used to prepare material for publication: *PLATON* (Spek, 2009 ▶) and *publCIF* (Westrip, 2010 ▶).

## Supplementary Material

Crystal structure: contains datablock(s) I. DOI: 10.1107/S160053681302744X/im2442sup1.cif

Structure factors: contains datablock(s) I. DOI: 10.1107/S160053681302744X/im2442Isup2.hkl

Supplementary material file. DOI: 10.1107/S160053681302744X/im2442Isup3.cml

Additional supplementary materials:  crystallographic information; 3D view; checkCIF report

## Figures and Tables

**Table 1:** Hydrogen-bond geometry (Å, °)

*D*—H⋯*A*	*D*—H	H⋯*A*	*D*⋯*A*	*D*—H⋯*A*
N1—H1⋯O2^i^	0.82	2.26	3.046 (2)	161
N2—H2⋯O1^ii^	0.88	1.99	2.862 (2)	174

Symmetry codes: (i) 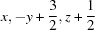; (ii) 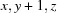.

**Fig. 1.**
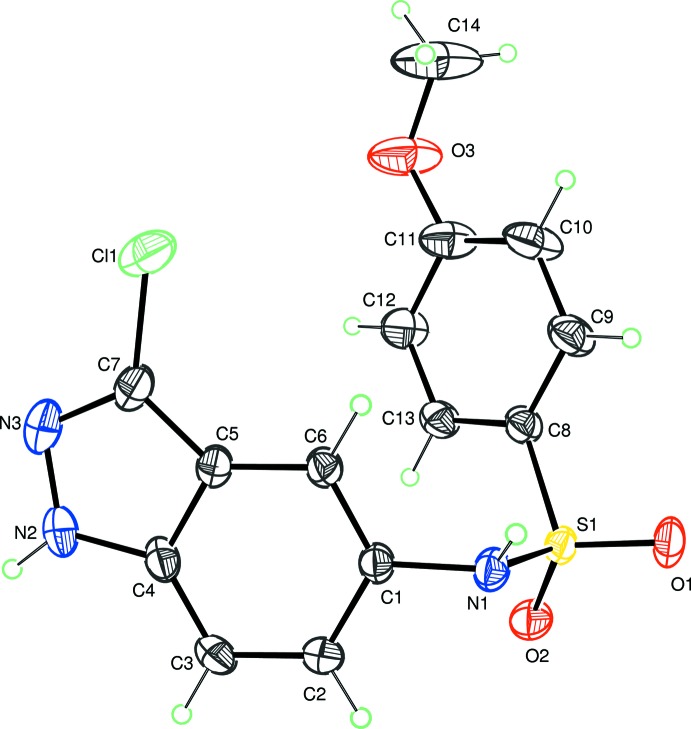
Molecular structure of the title compound with displacement ellipsoids drawn at the 50% probability level. H atoms are represented as small circles.

**Fig. 2.**
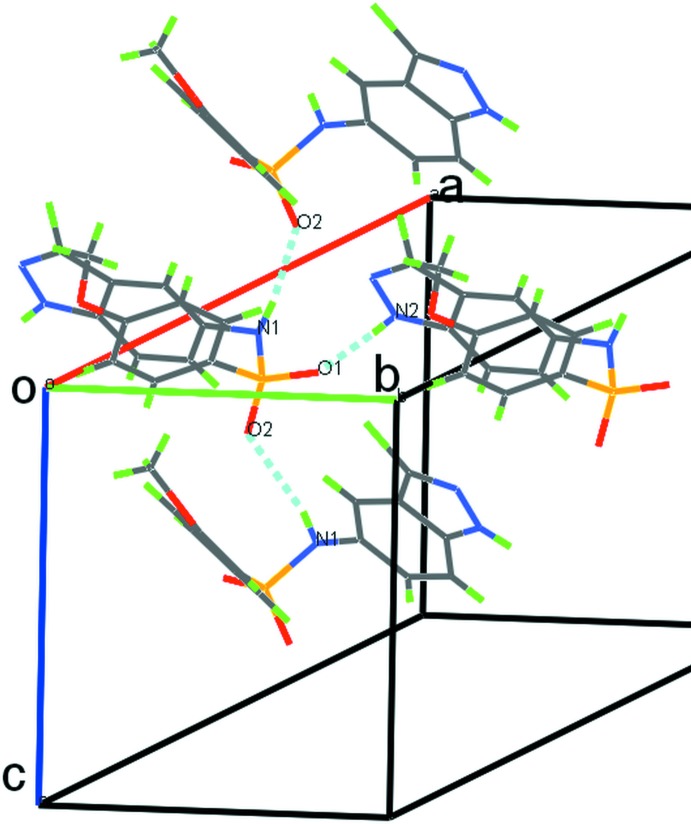
Partial crystal packing for the title compound showing N2—H2···O1 and N1—H1···O2 hydrogen bonds as dashed lines.

## supplementary crystallographic information

### 1. Comment

Sulfonamide derivatives are well known pharmaceutical agents since this group has been the main functional part in a vast number of drug structures due to stability and tolerance in human beings. These compounds exhibit a wide range of biological activities such as anticancer, anti-inflammatory, and antiviral functions (Abbassi *et al.*, 2012; Bouissane *et al.*, 2006; El-Sayed *et al.*, 2011; Mustafa *et al.*, 2012). The present work is a continuation of the investigation of sulfonamide derivatives published recently by our team (Abbassi *et al.*, 2013; Chicha *et al.*, 2013).

The molecule of *N*-(3-chloro-1*H*-indazol-5-yl)-4-methoxybenzenesulfonamide is built up from the 3-chloro-1*H*-indazol system (N2, N3, C1—C7, Cl) linked to the 4-methoxybenzenesulfonamide group as shown in Fig.1. The fused rings are slightly folded along the common edge and form a dihedral angle of 3.2 (1) °. Moreover, the dihedral angle between the mean plane through the indazole system and the plane through the atoms forming the benzene ring (C9—C14) is 30.75 (7)°.

In the crystal, molecules are interconnected by N1–H1···O2 and N2–H2···O1 hydrogen bonds forming a two-dimensional network parallel to (0 0 1) plane as shown in Fig.2 and Table 1.

### 2. Experimental

A mixture of 3-chloro-5-nitroindazole (1.22 mmol) and anhydrous SnCl_2_ (1.1 g, 6.1 mmol) in 25 ml of absolute ethanol was heated at 333 K for 6 h. After reduction, the starting material disappeared, and the solution was allowed to cool down. The pH was made slightly basic (pH 7–8) by addition of 5% aqueous potassium bicarbonate before extraction with ethyl acetate. The organic phase was washed with brine and dried over magnesium sulfate. The solvent was removed to afford the amine, which was immediately dissolved in pyridine (5 ml) and then reacted with 4-methoxybenzenesulfonyl chloride (1.25 mmol) at room temperature for 24 h. After the reaction mixture was concentrated in vacuum, the resulting residue was purified by flash chromatography (eluted with ethyl acetate: hexane 1:9, yield: 67%, mp: 445 K). The title compound was recrystallized from ethanol.

### 3. Refinement

H atoms were located in a difference map and treated as riding with C–H = 0.96 Å, C–H = 0.93 Å, and N–H = 0.89 Å for methyl, aromatic CH and NH, respectively. Thermal parameters were fixed at *U*_iso_(H) = 1.2 *U*_eq_ (aromatic, NH) and *U*_iso_(H) = 1.5 *U*_eq_ for methyl.

### Crystal data

**Table:**

C_14_H_12_ClN_3_O_3_S	*F*(000) = 696
*M**_r_* = 337.78	*D*_x_ = 1.465 Mg m^−^^3^
Monoclinic, *P*2_1_/*c*	Mo *K*α radiation, λ = 0.71073 Å
Hall symbol: -P 2ybc	Cell parameters from 4666 reflections
*a* = 16.1229 (5) Å	θ = 2.4–30.5°
*b* = 10.0562 (3) Å	µ = 0.40 mm^−^^1^
*c* = 9.7955 (2) Å	*T* = 296 K
β = 105.388 (1)°	Block, colourless
*V* = 1531.26 (7) Å^3^	0.42 × 0.35 × 0.28 mm
*Z* = 4	

### Data collection

**Table:**

Bruker X8 APEX diffractometer	4666 independent reflections
Radiation source: fine-focus sealed tube	3973 reflections with *I* > 2σ(*I*)
Graphite monochromator	*R*_int_ = 0.022
φ and ω scans	θ_max_ = 30.5°, θ_min_ = 2.4°
Absorption correction: multi-scan (*SADABS*; Bruker, 2009)	*h* = −23→20
*T*_min_ = 0.693, *T*_max_ = 0.747	*k* = −14→14
19939 measured reflections	*l* = −13→13

### Refinement

**Table:**

Refinement on *F*^2^	Primary atom site location: structure-invariant direct methods
Least-squares matrix: full	Secondary atom site location: difference Fourier map
*R*[*F*^2^ > 2σ(*F*^2^)] = 0.040	Hydrogen site location: inferred from neighbouring sites
*wR*(*F*^2^) = 0.117	H-atom parameters constrained
*S* = 1.03	*w* = 1/[σ^2^(*F*_o_^2^) + (0.0626*P*)^2^ + 0.5266*P*] where *P* = (*F*_o_^2^ + 2*F*_c_^2^)/3
4666 reflections	(Δ/σ)_max_ = 0.001
199 parameters	Δρ_max_ = 0.63 e Å^−^^3^
0 restraints	Δρ_min_ = −0.49 e Å^−^^3^

### Special details

**Table:**

Geometry. All s.u.'s (except the s.u. in the dihedral angle between two l.s. planes) are estimated using the full covariance matrix. The cell s.u.'s are taken into account individually in the estimation of s.u.'s in distances, angles and torsion angles; correlations between s.u.'s in cell parameters are only used when they are defined by crystal symmetry. An approximate (isotropic) treatment of cell s.u.'s is used for estimating s.u.'s involving l.s. planes.
Refinement. Refinement of *F*^2^ against all reflections. The weighted *R*-factor *wR* and goodness of fit *S* are based on *F*^2^, conventional *R*-factors *R* are based on *F*, with *F* set to zero for negative *F*^2^. The threshold expression of *F*^2^ > 2σ(*F*^2^) is used only for calculating *R*-factors(gt) *etc*. and is not relevant to the choice of reflections for refinement. *R*-factors based on *F*^2^ are statistically about twice as large as those based on *F*, and *R*- factors based on all data will be even larger.

### Fractional atomic coordinates and isotropic or equivalent isotropic displacement parameters (Å^2^)

**Table:**

	*x*	*y*	*z*	*U*_iso_*/*U*_eq_	
C4	0.60822 (8)	0.97963 (12)	−0.02865 (13)	0.0268 (2)	
C5	0.56209 (8)	1.06572 (14)	−0.13562 (15)	0.0336 (3)	
H5	0.5231	1.0301	−0.2147	0.040*	
C6	0.57361 (10)	1.20157 (14)	−0.12547 (17)	0.0386 (3)	
H6	0.5432	1.2585	−0.1960	0.046*	
C7	0.63294 (9)	1.24992 (12)	−0.00465 (16)	0.0347 (3)	
C2	0.67936 (9)	1.16423 (13)	0.10154 (14)	0.0306 (3)	
C3	0.66674 (8)	1.02644 (12)	0.09022 (13)	0.0286 (2)	
H3	0.6968	0.9690	0.1604	0.034*	
C1	0.73489 (11)	1.25131 (14)	0.19788 (17)	0.0400 (3)	
C8	0.76161 (9)	0.78982 (13)	−0.03973 (14)	0.0305 (2)	
C9	0.80235 (10)	0.71057 (16)	0.07419 (17)	0.0427 (3)	
H9	0.7756	0.6340	0.0946	0.051*	
C10	0.88322 (11)	0.7460 (2)	0.1577 (2)	0.0533 (5)	
H10	0.9112	0.6926	0.2334	0.064*	
C11	0.92203 (10)	0.8613 (2)	0.12745 (18)	0.0497 (4)	
C12	0.88199 (10)	0.93915 (17)	0.01121 (17)	0.0436 (3)	
H12	0.9094	1.0146	−0.0105	0.052*	
C13	0.80182 (9)	0.90444 (14)	−0.07167 (15)	0.0348 (3)	
H13	0.7745	0.9570	−0.1486	0.042*	
C14	1.03882 (16)	0.8432 (4)	0.3356 (3)	0.1044 (12)	
H14A	1.0928	0.8853	0.3792	0.157*	
H14B	1.0484	0.7512	0.3185	0.157*	
H14C	1.0017	0.8498	0.3974	0.157*	
N1	0.59374 (7)	0.83818 (10)	−0.04781 (12)	0.0298 (2)	
H1	0.5932	0.7982	0.0253	0.036*	
N2	0.66110 (10)	1.37511 (12)	0.03591 (17)	0.0486 (3)	
H2	0.6490	1.4494	−0.0128	0.058*	
N3	0.72483 (10)	1.37633 (13)	0.15800 (16)	0.0497 (3)	
O1	0.63534 (8)	0.62011 (10)	−0.11826 (12)	0.0436 (3)	
O2	0.63874 (7)	0.81515 (10)	−0.26973 (10)	0.0378 (2)	
O3	0.99969 (9)	0.9074 (2)	0.20519 (17)	0.0789 (5)	
S1	0.65443 (2)	0.75832 (3)	−0.13090 (3)	0.02874 (9)	
Cl1	0.80966 (4)	1.20719 (5)	0.34939 (5)	0.06387 (16)	

### Atomic displacement parameters (Å^2^)

**Table:**

	*U*^11^	*U*^22^	*U*^33^	*U*^12^	*U*^13^	*U*^23^
C4	0.0299 (5)	0.0219 (5)	0.0284 (5)	−0.0007 (4)	0.0075 (4)	−0.0003 (4)
C5	0.0305 (6)	0.0314 (6)	0.0339 (6)	0.0016 (5)	−0.0001 (5)	0.0017 (5)
C6	0.0366 (7)	0.0296 (6)	0.0441 (8)	0.0063 (5)	0.0011 (6)	0.0087 (5)
C7	0.0370 (7)	0.0217 (5)	0.0443 (7)	0.0024 (5)	0.0087 (6)	0.0009 (5)
C2	0.0354 (6)	0.0242 (5)	0.0311 (6)	−0.0014 (5)	0.0067 (5)	−0.0032 (4)
C3	0.0358 (6)	0.0228 (5)	0.0259 (5)	0.0005 (4)	0.0058 (5)	0.0017 (4)
C1	0.0471 (8)	0.0322 (7)	0.0372 (7)	−0.0059 (6)	0.0049 (6)	−0.0085 (5)
C8	0.0352 (6)	0.0283 (6)	0.0269 (6)	0.0033 (5)	0.0065 (5)	0.0024 (4)
C9	0.0423 (8)	0.0406 (7)	0.0428 (8)	0.0026 (6)	0.0068 (6)	0.0160 (6)
C10	0.0381 (8)	0.0683 (12)	0.0478 (9)	0.0044 (7)	0.0011 (7)	0.0286 (8)
C11	0.0311 (7)	0.0727 (12)	0.0421 (8)	−0.0022 (7)	0.0040 (6)	0.0134 (8)
C12	0.0372 (7)	0.0485 (8)	0.0436 (8)	−0.0060 (6)	0.0080 (6)	0.0109 (7)
C13	0.0381 (7)	0.0336 (6)	0.0313 (6)	0.0015 (5)	0.0066 (5)	0.0073 (5)
C14	0.0523 (12)	0.175 (3)	0.0664 (15)	−0.0201 (16)	−0.0193 (11)	0.0445 (18)
N1	0.0363 (5)	0.0232 (5)	0.0295 (5)	−0.0051 (4)	0.0081 (4)	−0.0004 (4)
N2	0.0575 (8)	0.0211 (5)	0.0617 (9)	0.0005 (5)	0.0065 (7)	0.0009 (5)
N3	0.0595 (9)	0.0294 (6)	0.0568 (9)	−0.0080 (6)	0.0091 (7)	−0.0109 (6)
O1	0.0615 (7)	0.0206 (4)	0.0443 (6)	−0.0064 (4)	0.0063 (5)	−0.0047 (4)
O2	0.0491 (6)	0.0382 (5)	0.0226 (4)	−0.0019 (4)	0.0033 (4)	0.0000 (4)
O3	0.0394 (6)	0.1184 (14)	0.0647 (9)	−0.0214 (8)	−0.0107 (6)	0.0322 (9)
S1	0.03872 (17)	0.02109 (14)	0.02355 (15)	−0.00219 (11)	0.00327 (12)	−0.00259 (10)
Cl1	0.0721 (3)	0.0615 (3)	0.0427 (2)	−0.0113 (2)	−0.0115 (2)	−0.00649 (19)

### Geometric parameters (Å, º)

**Table:**

C4—C3	1.3728 (17)	C9—H9	0.9300
C4—C5	1.4096 (17)	C10—C11	1.387 (3)
C4—N1	1.4456 (15)	C10—H10	0.9300
C5—C6	1.3787 (19)	C11—O3	1.362 (2)
C5—H5	0.9300	C11—C12	1.391 (2)
C6—C7	1.397 (2)	C12—C13	1.377 (2)
C6—H6	0.9300	C12—H12	0.9300
C7—N2	1.3613 (17)	C13—H13	0.9300
C7—C2	1.4041 (19)	C14—O3	1.421 (3)
C2—C3	1.4006 (16)	C14—H14A	0.9600
C2—C1	1.4180 (18)	C14—H14B	0.9600
C3—H3	0.9300	C14—H14C	0.9600
C1—N3	1.314 (2)	N1—S1	1.6393 (12)
C1—Cl1	1.7038 (17)	N1—H1	0.8235
C8—C9	1.3866 (19)	N2—N3	1.354 (2)
C8—C13	1.3980 (19)	N2—H2	0.8799
C8—S1	1.7507 (14)	O1—S1	1.4359 (10)
C9—C10	1.389 (2)	O2—S1	1.4344 (10)
			
C3—C4—C5	121.88 (11)	O3—C11—C10	124.39 (15)
C3—C4—N1	119.90 (11)	O3—C11—C12	115.10 (16)
C5—C4—N1	118.21 (11)	C10—C11—C12	120.50 (15)
C6—C5—C4	121.37 (12)	C13—C12—C11	119.94 (14)
C6—C5—H5	119.3	C13—C12—H12	120.0
C4—C5—H5	119.3	C11—C12—H12	120.0
C5—C6—C7	117.05 (12)	C12—C13—C8	119.72 (13)
C5—C6—H6	121.5	C12—C13—H13	120.1
C7—C6—H6	121.5	C8—C13—H13	120.1
N2—C7—C6	132.03 (13)	O3—C14—H14A	109.5
N2—C7—C2	106.28 (13)	O3—C14—H14B	109.5
C6—C7—C2	121.64 (12)	H14A—C14—H14B	109.5
C3—C2—C7	120.72 (12)	O3—C14—H14C	109.5
C3—C2—C1	135.76 (13)	H14A—C14—H14C	109.5
C7—C2—C1	103.44 (12)	H14B—C14—H14C	109.5
C4—C3—C2	117.33 (11)	C4—N1—S1	116.71 (9)
C4—C3—H3	121.3	C4—N1—H1	113.9
C2—C3—H3	121.3	S1—N1—H1	109.7
N3—C1—C2	112.75 (14)	N3—N2—C7	112.47 (13)
N3—C1—Cl1	120.74 (12)	N3—N2—H2	118.6
C2—C1—Cl1	126.50 (12)	C7—N2—H2	128.2
C9—C8—C13	120.34 (13)	C1—N3—N2	105.00 (12)
C9—C8—S1	119.95 (11)	C11—O3—C14	118.12 (18)
C13—C8—S1	119.25 (10)	O2—S1—O1	118.65 (6)
C8—C9—C10	119.78 (15)	O2—S1—N1	107.29 (6)
C8—C9—H9	120.1	O1—S1—N1	105.16 (6)
C10—C9—H9	120.1	O2—S1—C8	108.27 (6)
C11—C10—C9	119.68 (14)	O1—S1—C8	109.65 (7)
C11—C10—H10	120.2	N1—S1—C8	107.25 (6)
C9—C10—H10	120.2		

### Hydrogen-bond geometry (Å, º)

**Table:**

*D*—H···*A*	*D*—H	H···*A*	*D*···*A*	*D*—H···*A*
N1—H1···O2^i^	0.82	2.26	3.046 (2)	161
N2—H2···O1^ii^	0.88	1.99	2.862 (2)	174

Symmetry codes: (i) *x*, −*y*+3/2, *z*+1/2; (ii) *x*, *y*+1, *z*.
